# Impact of the COVID-19 pandemic on commencement of psychotropic medications in Northern Ireland: a population-wide, administrative data linkage study.

**DOI:** 10.23889/ijpds.v7i3.1915

**Published:** 2022-08-25

**Authors:** Lisa Kent, Dermot O'Reilly, Aideen Maguire

**Affiliations:** 1 Administrative Data Research Centre Northern Ireland (ADRC-NI), Queen’s University, Belfast; 2 Centre for Public Health, Queen's University Belfast

## Objectives

This study aimed to explore changes in commencement of psychotropic medications in first 20 months of the pandemic and associated restrictions in Northern Ireland (NI).

## Approach

Antidepressant, anxiolytic, hypnotic and antipsychotic medications dispensed in all community pharmacies in NI (Jan-2012 to Oct-2021, Enhanced Prescribing Database) were linked to sociodemographic data (National Health Application and Infrastructure Services) for everyone alive and resident in NI aged ≥10years. Commencement of new medication was identified on a rolling monthly basis as a dispensation in a given month but not in the previous 24 months. Auto Regressive Integrated Moving Average (ARIMA) models were trained taking trends and seasonal effects into consideration. Monthly forecasts were compared to actual numbers, at population level and within sociodemographic groups (gender, age, rurality, living-alone, deprivation).

## Results

There were consistent increased numbers of individuals commencing antipsychotic medications in the group aged ≥65years, with observed to expected ratio ranging from 1.12 to 2.1. Commencement of hypnotics was decreased throughout the study in individuals aged <18years (OER ranged from 0.28 to 0.70) but remained as expected for other sociodemographic groups. Across all sociodemographic groups, commencement of antidepressants decreased initially (Mar-May 2020 population-level OER ranged from 0.61 to 0.79) and in Jan 2021 (population-level OER 0.78) corresponding with stricter stay at home restrictions but resumed the expected trend outside of these periods. There were no obvious deviations from expected trends in commencement of anxiolytics.

## Conclusion

There was a clear impact on older people with regards commencement of antipsychotic medications throughout the pandemic. Hypnotic commencement in children was lower than expected throughout the pandemic, which may reflect reduced need or reduced access to specialist paediatric services. (NHS-REC:20/YH/0254; Data sourced from the Honest Broker Service)

